# Spleen tyrosine kinase inhibitors disrupt human neutrophil swarming and antifungal functions

**DOI:** 10.1128/spectrum.02549-21

**Published:** 2024-11-27

**Authors:** Alex Hopke, Adam L. Viens, Natalie J. Alexander, Seok Joon Mun, Michael K. Mansour, Daniel Irimia

**Affiliations:** 1 BioMEMS Resource Center, Massachusetts General Hospital, Boston, Massachusetts, USA; 2 Harvard Medical School, Boston, Massachusetts, USA; 3 Shriners Hospital for Children, Boston, Massachusetts, USA; 4 Division of Infectious Diseases, Massachusetts General Hospital, Boston, Massachusetts, USA; Indian Institute of Science Bangalore, Bangalore, Karnataka, India

**Keywords:** swarming, neutrophil, SYK inhibitor, JNK signaling, PI3Ky signaling, GCSF

## Abstract

Neutrophils communicate with one another and amplify their destructive power through swarming, a collective process that synchronizes the activities of multiple neutrophils against one target. The sequence of activities contributing to swarming against clusters of fungi has been recently uncovered. However, the molecular signals controlling the neutrophils’ activities during the swarming process are just emerging. Here, we report that spleen tyrosine kinase (SYK) inhibitors severely impair neutrophil swarming responses, resulting in the complete loss of fungal restriction. These findings are enabled by a microscale platform to probe the biology of human neutrophils swarming against uniformly sized clusters of growing *Candida albicans*, a representative opportunistic fungal pathogen. We take advantage of the ability to monitor large arrays of swarms and quantify the effect of multiple chemical inhibitors on different phases of human neutrophil swarming. We show that inhibitors that interfere with PI3Ky signaling disrupt the regulation of the initiation of swarming, while the activation of JNK signaling is essential for the activation of biochemical antifungal functions. Furthermore, we reveal that granulocyte colony-stimulating factors (GCSF and GM-CSF) can partially rescue the antifungal functions of neutrophils exposed to SYK inhibitors. These findings advance our understanding of neutrophil swarming biology in humans and lay the foundation for novel therapeutics that may restore neutrophil function during immunosuppression.

**IMPORTANCE:**

Neutrophils can amplify their destructive power through swarming, a crucial process against large targets that individual neutrophils cannot destroy. However, the molecular mechanisms controlling this process are just emerging. Here, we leveraged microscale tools to probe the biology of swarming against fungi. We used multiple chemical inhibitors and mapped SYK, PI3Ky, and JNK signaling roles during human neutrophil swarming against fungal clusters of *Candida albicans*. We also found that treating human neutrophils with GCSF and GM-CSF rescues some neutrophil antifungal function during SYK inhibition. These findings advance our understanding of swarming biology in humans while laying the foundation for developing therapeutics that enhance neutrophil function during immunosuppression.

## INTRODUCTION

Fungal infections are a considerable scourge with high mortality in immunocompromised patients (1, 2). Neutrophils play a significant role in antifungal defense, and recently, a novel behavior of neutrophils, termed swarming, has begun to be characterized with the help of specially designed *in vitro* arrays and *in vivo* animal models (3–7). In swarming, neutrophils cooperatively promote their recruitment and achieve exponentially fast accumulations at the sites of inflammatory or microbial stimuli (4, 8). Swarming may be particularly relevant to the defense against fungi, when hyphae and other enlarged morphologies prevent individual neutrophils from phagocytosing and clearing the microbes (7, 9).

This efficient response against fungi relies on a sequence of antimicrobial mechanisms that start with the rapid migration of neutrophils toward the fungi clusters, are enhanced by the release of leukotriene B_4_ (LTB_4_) and other chemoattractants by the pioneer neutrophils, and are followed by the generation of reactive oxygen species, the release of proteases, and the formation of neutrophil extracellular traps (10, 11). However, the molecular mechanisms that allow neutrophils to discriminate between situations that elicit a swarming response and those that would not and then signal these decisions remain unclear. Swarming and its corresponding antimicrobial strategies are likely deployed after receptors, like C-type lectins, recognize elements of the fungal cell wall, and set off signaling cascades (12, 13). Many of these signaling cascades utilize kinases, including spleen tyrosine kinase (SYK) and adapters, such as CARD9, which are essential for effective antifungal responses by both innate and adaptive immunity (12, 14–16).

SYK signaling in neutrophils is critical for the killing of both opsonized and unopsonized *Candida albicans* in traditional assays (16, 17). SYK also plays a critical role in the swarming response of mouse neutrophils, though the rest of the signaling pathway remains unclear (18). A more complete elucidation of the role played by SYK in mediating swarming will be required to fully understand the role of swarming in antifungal defense.

While critical for antifungal immunity, the highly inflammatory actions of neutrophils can also result in severe damage to the host and have been implicated in several autoimmune and inflammatory diseases (19–22). SYK signaling, for example, has been implicated in certain forms of cancer, rheumatoid arthritis, and other inflammatory diseases (14, 23). Inhibition of these SYK-mediated inflammatory pathways has therefore been investigated as a therapeutic strategy for these diseases with promising results (14, 24–26). However, due to the critical role of SYK signaling in neutrophil antifungal defense, these patients are likely to be more susceptible to infectious complications, similar to those undergoing treatments with other anti-inflammatory agents (2, 27). To develop interventions that can minimize this risk, it will be critical to fully understand the pathways contributing to and regulating the neutrophil antimicrobial functions, including those that govern swarming.

Here, we leverage our unique swarming array tool to probe the molecular pathways regulating swarming antifungal function. We build upon our previous work examining the role of SYK in mice to confirm the importance of SYK signaling in human swarming responses and we then begin to explore the role of potential downstream molecular mediators, including PLC, c-Fos/AP-1, JNK, and PI3Kγ. Of practical importance, we found that we could restore some neutrophil antifungal function during SYK inhibition with the cytokines GM-CSF or GCSF. Taken together, our results contribute to and expand our molecular understanding of the pathways involved in the swarming response to fungi.

## MATERIALS AND METHODS

### Micro-organism culture

*C. albicans* (SC5314), constitutively expressing a far-red fluorescent protein, was inoculated into 5 mL fresh liquid YPD media and grown with shaking overnight at 30°C (28).

### Neutrophil isolation

Fresh peripheral blood samples from healthy volunteers were collected in 10 mL EDTA tubes (Research Blood Components LLC, Allston, MA) and utilized within 6 h after the blood draw. Protocols were approved by the institutional review board at Massachusetts General Hospital. Neutrophils were isolated using the EasySep Direct Human Neutrophil isolation kit per the manufacturer’s protocol (STEMCELL Technologies). Isolated neutrophils were stained with Hoechst (ThermoFisher Scientific) and re-suspended in IMDM with 20% FBS (ThermoFisher Scientific) before use for swarming assays.

### Array printing

Swarming arrays of live *C. albicans* were prepared as described previously (6, 7). We used a microprinting platform (Picospotter PolyPico Galway, Ireland) to print a solution of 0.1% poly-l-lysine (Sigma-Aldrich) with ZETAG in 100 µm diameter spots. For experiments, we printed eight-by-eight arrays in a 16-well format on ultra-clean glass slides (Fisher Scientific). Slides were dried on a heat block at 45°C for 2 h and left at room temperature until used in experiments.

### Target patterning

Sixteen-well ProPlate wells (Grace Bio-labs) were attached to glass slides with the printed arrays. An overnight culture of live *C. albicans* yeast was re-suspended in dH_2_O, then 50 µL was added to each well and incubated with rocking for 5 min. Following incubation, wells were thoroughly washed with phosphate-buffered saline to remove unbound targets from the glass surface. Wells were screened to ensure appropriate patterning of targets onto the spots with minimal non-specific binding before use.

### Swarming experiments

All swarming experiments were conducted using a fully automated Nikon TiE microscope with a temperature-controlled incubator set at 37°C. Time-lapse imaging was conducted using a 10× Plan Fluor Ph1 DLL (NA = 0.3) lens, and endpoint images were taken with a 2× Plan Apo (NA = 0.10) lens. Images were acquired every 10 min for 16 h. Up to 16 targets were imaged for each condition. Swarming targets to be observed during time-lapse were selected and saved using the multipoint function in NIS elements prior to the loading of neutrophils. After isolation, 500,000 purified neutrophils were added to each well. Before launching the experiment, all selected points were optimized using the Nikon Perfect Focus system. Neutrophils were pre-incubated with the inhibitor or appropriately matched vehicle control for 30 min before use.

### NETosis visualization

We visualized DNA release by adding Sytox green to the media at 500 nM (ThermoFisher Scientific). Hoechst staining was also leveraged to visualize and track individual neutrophil nuclei as they progressed from condensed to diffuse while releasing NETs on the target fungal clusters over time.

### Chemical inhibitors

The SYK inhibitors R406, RO9021, and PRT-060318 2HCL (Selleck Chemicals) were used for SYK inhibition (18). R406 and RO9021 were used at a final concentration of 10 µM, and PRT-060318 2HCL was used at 2 µM. Other targets were inhibited with Edelfosine (Phospholipase C), AS605240 (PI3Kγ), T5224 (c-Fos/AP-1), or SP600125 (JNK) at a final concentration of 10 µM (29–33). All chemical inhibitors were accompanied and compared to the appropriate vehicle control (DMSO for R406, RO9021, AS605240, T5224, and SP600125, water for PRT-060318 2HCL and Edelfosine). GM-CSF was used at a concentration of 0.2 ng/mL, and GCSF was used at a concentration of 300 ng/mL (7). Neutrophils were preincubated with the indicated chemical or vehicle for 30 min prior to addition to the swarming arrays. Concentrations were selected based on our previous published work, published literature surveys, and manufacturer recommendations (7, 18, 29–36).

### Reactive oxygen species production assay

Healthy donor neutrophils were plated in a white-walled 96-well plate (Grenier Bio-One, Monroe, NC) at 1 × 10^5^ cells per well in complete medium. With the plate on ice, heat-killed *C. albicans* hyphae were added at a multiplicity of infection (MOI) of 20. SP600125 was plated at desired concentrations, and the lucigenin solution was added immediately for a final concentration of 15 µM lucigenin (Enzo Life Sciences, Farmingdale, NY). Luminescence was measured every 5 min for 4 h in a SpectraMax i3x reader (Molecular Devices, San Jose, CA) at 37°C. The sum total of the arbitrary luminescence units over the 4-h period represents the results. Statistical analysis was performed using GraphPad Prism, applying an unpaired *t* test to the results. The results represent the mean ± SEM of one experiment and are representative of three independent experiments.

### Neutrophil killing assay

Neutrophil killing assays were conducted as described (18, 37). Briefly, isolated primary human neutrophils were plated at 5 × 10^4^ cells per well in a complete RPMI medium (RPMI 1640 with 2 mM l-glutamine, 10% heat-inactivated fetal bovine serum, and 1% penicillin-streptomycin, ThermoFisher Scientific, Waltham, MA) in a 96-well clear-bottom plate. Neutrophils were simultaneously primed with either GCSF (at a concentration of 300 ng/mL) or GM-CSF (at a concentration of 0.2 ng/mL), and pre-incubated with either R406 or RO9021 (at a concentration of 10 µM), for 30 min at 37°C and 5% CO_2_. After pretreatment, *C. albicans* at a MOI of 10 were co-incubated with neutrophils for 2 h. Following incubation, mammalian cells were lysed with 4× NP-40 (Nonidet P40 purchased from American Bioanalytical, Natick, MA), and each well-received an addition of optimized yeast growth media, MOPS-RPMI (RPMI 1640 containing 2% glucose and 0.165 M MOPS, buffered at pH = 7), to supplement *Candida* growth. Finally, 10% PrestoBlue Cell Viability Reagent (ThermoFisher Scientific) was added to each well. Fluorescence was read every 30 min for 18 h at 30°C using a SpectraMax i3x plate reader (Molecular Devices, Sunnyvale, CA). Fluorescence was plotted versus time, and the time to the mid-curve (inflection point) was determined using GraphPad Prism software. Results were reported as the percent of pathogens killed by the neutrophils (percent killing).

### Image analysis

Fungal growth and restriction were analyzed in four ways: total area of fungal growth, growth over time by far-red fluorescence in time-lapse microscopy, time taken for *C. albicans* yeast to germinate, and time taken for *C. albicans* hyphae to escape the neutrophil swarm. Area analysis was performed manually by outlining the areas of fungal growth in FIJI (FIJI is just ImageJ, NIH) software at the end of the assay (16 h). For the area of fungal growth measurements, a combination of brightfield and fluorescent channels was used. *C. albicans* used in experiments were always the fluorescent WT-FarRed670 strain (28). We combined the appropriate fluorescent channel with the brightfield image to be sure we included any escaped fungal elements, for example, lone hyphae that may not appear well in the fluorescent channel. For scoring of germination, we observed the fluorescent fungi in each swarm, determining the frame when germination becomes visible. For scoring of hyphal escape, we observed the fluorescent fungi in each swarm via time-lapse and determined the time when hyphae first breached the containment of the swarm (extends beyond the area of neutrophils). For *C. albican*s, in the absence of neutrophils, the escape was defined as growth beyond the area of an average swarm. For fungal growth over time, fluorescent intensity profiles were generated by defining regions of interest and using the time measurement option for far-red fluorescence in FIJI software. Fluorescent intensity profiles were also used to examine swarming dynamics via Hoechst-stained neutrophils and DNA release via Sytox Green staining.

### Statistics

Data were tested for normality using a D'Agostino-Pearson omnibus normality test. Normally distributed data were analyzed with one-way ANOVA with Tukey’s post-test. Non-normally distributed data were analyzed with Kruskal-Wallis tests. Statistical significance was considered for *P* < 0.05. All statistics were conducted using GraphPad Prism software.

## RESULTS

SYK is well established to have a critical role in the molecular signaling pathways facilitating antifungal responses (12–15). Our earlier results have expanded that role to include mouse neutrophil swarming (18). Here, we sought to elucidate further the molecular mechanisms involved in the regulation of neutrophil swarming against fungi by expanding the study to human neutrophils and leveraging our *in vitro* swarming assay, which consists of arrays of live *C. albicans* clusters on glass slides. We show that chemical inhibition of SYK abrogates human neutrophil swarming against *C. albicans* clusters. We further probed the role of downstream signaling, including PI3Ky and JNK, during this process in human cells.

**Fig 1:**
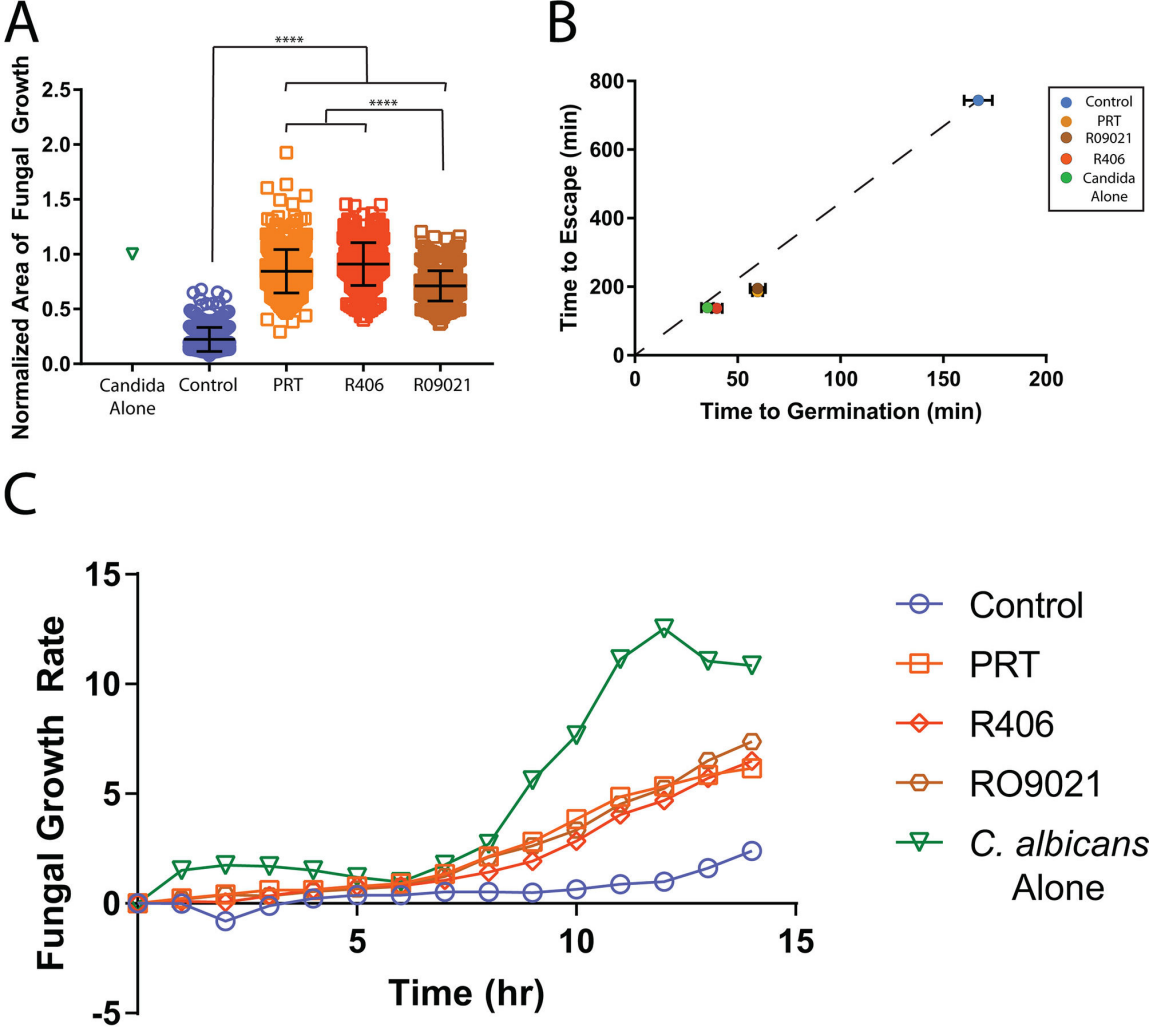
Fungal growth in the presence of neutrophil swarms and SYK inhibititors. Treatment of human neutrophils with SYK inhibitors results in significant loss of fungal restriction. Human neutrophils were treated with the indicated SYK inhibitor or with an appropriate vehicle control before being added to wells. (**A**) The area covered by *C. albicans* after 16 h, normalized to the growth of *C. albicans* alone for the same time, was quantified. *N* ≥ 414 swarms across six independent donors. (**B**) The time it took for *C. albicans* yeast to germinate into hyphae and for those hyphae to escape the neutrophil swarm area were quantified and plotted. *N* ≥ 80 swarms across five independent donors. (**C**) The average fluorescent intensity of *C. albicans* (which expresses a far-red fluorescent protein) was quantified over the time-lapse. The approximate slope of the fluorescent intensity plot for each time point was found to determine the rate of fungal growth. *N* = 16 swarms from a representative donor. Error bars represent the standard deviation for panel A or the standard error for panel B. ****P ≤ 0.0001.

When healthy donor control neutrophils encounter large clusters of yeast in our arrays, they form robust swarms, following an exponential recruitment phase. During the early phases of swarming, the neutrophils directly attack *C. albicans* utilizing classical antimicrobial strategies, including the action of MPO and the generation of reactive oxygen species (ROS) inside the swarm (7). Early neutrophil swarming significantly delays *C. albicans* yeast germination into hyphae. Moreover, the early neutrophil swarms restrict *C. albicans* growth to spaces at least four times smaller than the area *C. albicans* would grow to in the absence of neutrophils (Fig. 1A; Fig. S1; Table S1). Neutrophil swarms also interfere with the growth of *C. albicans* at later stages through the release of NETs, around 4 h after swarming initiation. NETs quickly cover the fungal clusters within the swarm and block the growth of surviving hyphae (Fig. 1B; Fig. S1; Table S1). These observations agree with previous results using healthy donor neutrophils (7) and serve as a reference for the effect of various inhibitors and cytokines.

**Fig 2:**
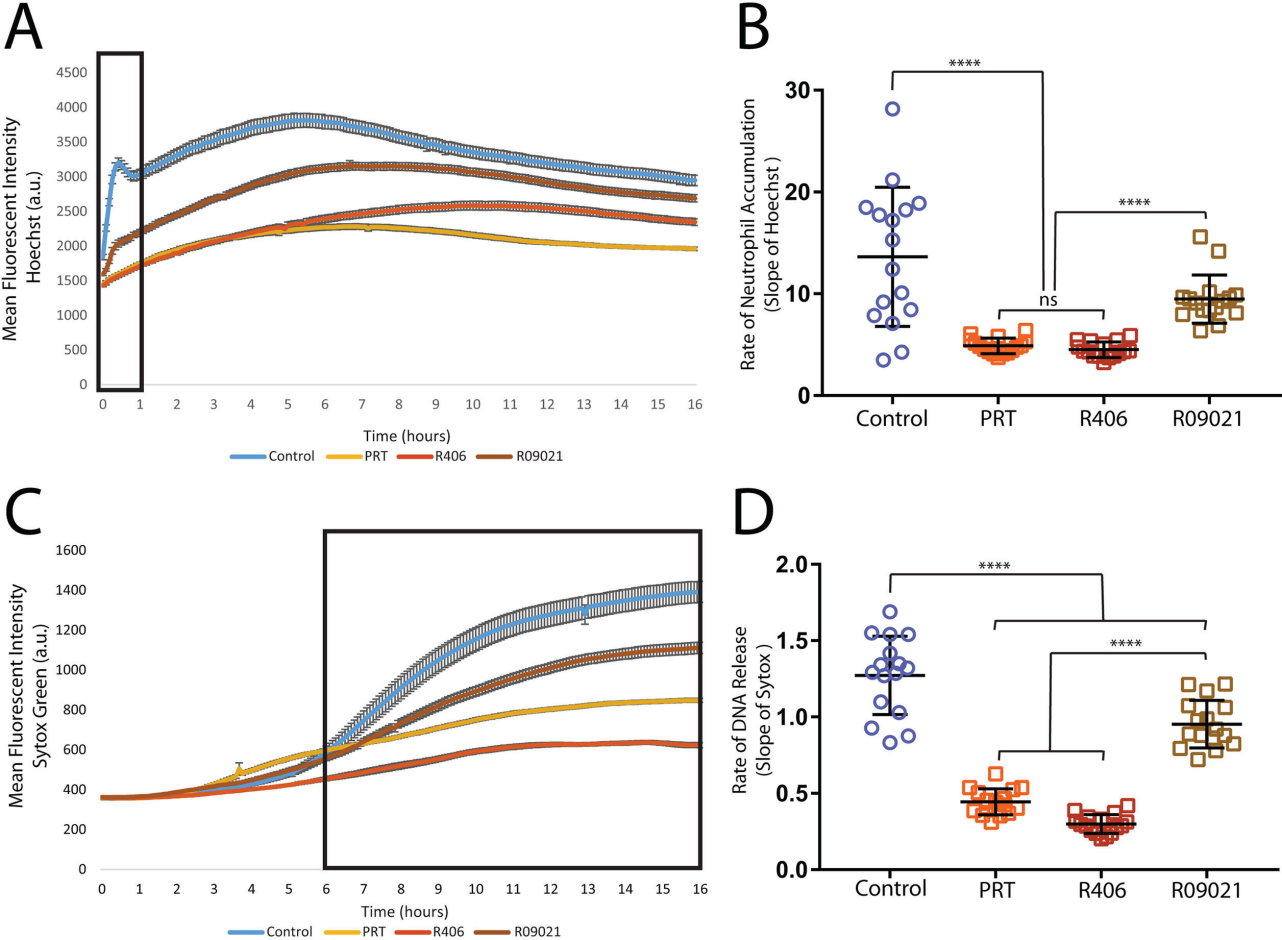
Neutrophil swarming function is disrupted by SYK inhibition. (**A**) Disruption of SYK signaling inhibits neutrophil swarming and NET release. Human neutrophils were treated with the indicated SYK inhibitor or appropriate vehicle control. Neutrophils were stained with Hoechst and the average fluorescent intensity was quantified at the target as an indicator of neutrophils’ swarm intensity. (**B**) The slope of these intensity plots was determined over the first hour. (**C**) The average fluorescent intensity of sytox green staining at the target was quantified over the time-lapse as an indicator of DNA release. (**D**) The rate of DNA release between 5 and 13 h is reduced in the presence of SYK inhibitors. *N* = 16 swarms from a representative donor for all panels. Error bars represent the standard deviation for panels B and D or the standard error for panels A and C. ****P ≤ 0.0001.

When we use SYK inhibitors R406, RO9021, and PRT-060318, we observe a significant reduction in the ability of neutrophils to restrict *C. albicans* growth. By the end of the assay, we observe four times more fungal growth in treated than untreated neutrophils (Fig. 1A; Fig. S1; Table S1). We also observe that *C. albicans* yeast germinate into hyphae and escape neutrophil swarms faster than is seen with untreated or vehicle control-treated neutrophils (Fig. 1B; Table S1). Overall, treatment with SYK inhibitors results in significantly increased fungal growth rates compared to the control swarms, with the largest differences occurring after 8 h and beyond (Fig. 1C). During SYK inhibitor treatment, neutrophils do not display an exponential recruitment phase in the first hour, suggesting that the positive feedback between neutrophils typically seen during swarming is absent (Fig. 2A and B; Table S2).

The early failure of neutrophil recruitment to the swarm in the presence of SYK inhibitors contributes to the increased fungal growth seen later in the assay (Fig. 1 and 2). For example, DNA release was also significantly reduced during treatment with all three SYK inhibitors, and this is likely related to the lower number of neutrophils recruited to the fungal cluster (Fig. 2C and D; Fig. S1; Table S2). While immunostaining confirmed the presence of citrullinated histone H3 in healthy donor NETs, identifying them as “true NETs,” the low number of cells present at targets following SYK inhibition made immunostaining difficult (7). We, therefore, used a combination of methods to further quantify NETs in these SYK-inhibited conditions. First, we quantified the number of visible, condensed nuclei present in the swarm (or on the *C. albicans* target when no swarm occurred) over time, and we also included sytox green, a fluorescent probe that binds external DNA and, therefore, stains released NETs. By quantifying condensed nuclei over time, we can track changes to their number as they decondense and expand in a manner characteristic of NET release (38). The healthy control swarms show the same trend we found previously, with a progressive loss of condensed nuclei beginning after 4 h into the assay, which lines up with the beginning of sytox green staining and the timing of the classical NET release (Fig. S2a through c) (7). Taken together, these analyses indicate that NETs are being released properly in our swarming assay. Neutrophils treated with SYK inhibitors R406 or PRT-060318 displayed similar trends, though to a lesser extent in keeping with their lack of neutrophil recruitment to targets (Fig S2b and c).

While all three SYK inhibitors result in significant defects to neutrophil antifungal activity and disruptions to swarming dynamics, we could measure several differences among the inhibitors (Fig. 1 and 2; Fig. S1; Tables S1 and S2). Of particular note, neutrophils treated with RO9021 managed to restrict overall fungal growth better than PRT or R406. However, all three inhibitors still induce a significant defect compared to control (Fig. 1A; Table S1). RO9021-treated neutrophils are also recruited to a significantly higher level and released more NETs than either PRT or R406-treated cells (Fig. 2; Table S2). Furthermore, neutrophils treated with R406 experience a more pronounced deficit in the ability to restrict *C. albicans*, with significantly lower times to germination and hyphal escape than either PRT or RO9021 (Fig. 1B; Table S1). In keeping with this, R406-treated neutrophils also displayed the lowest DNA release profile of all three SYK inhibitors (Fig. 2C and D; Table S2). Despite these differences, the overall phenotype that treatment with each of these SYK inhibitors induces, that of disrupted swarming and compromised antifungal efficacy, is aligned with each other and with findings in mouse neutrophils, suggesting that SYK plays a critical role in swarming in human neutrophils.

**Fig 3:**
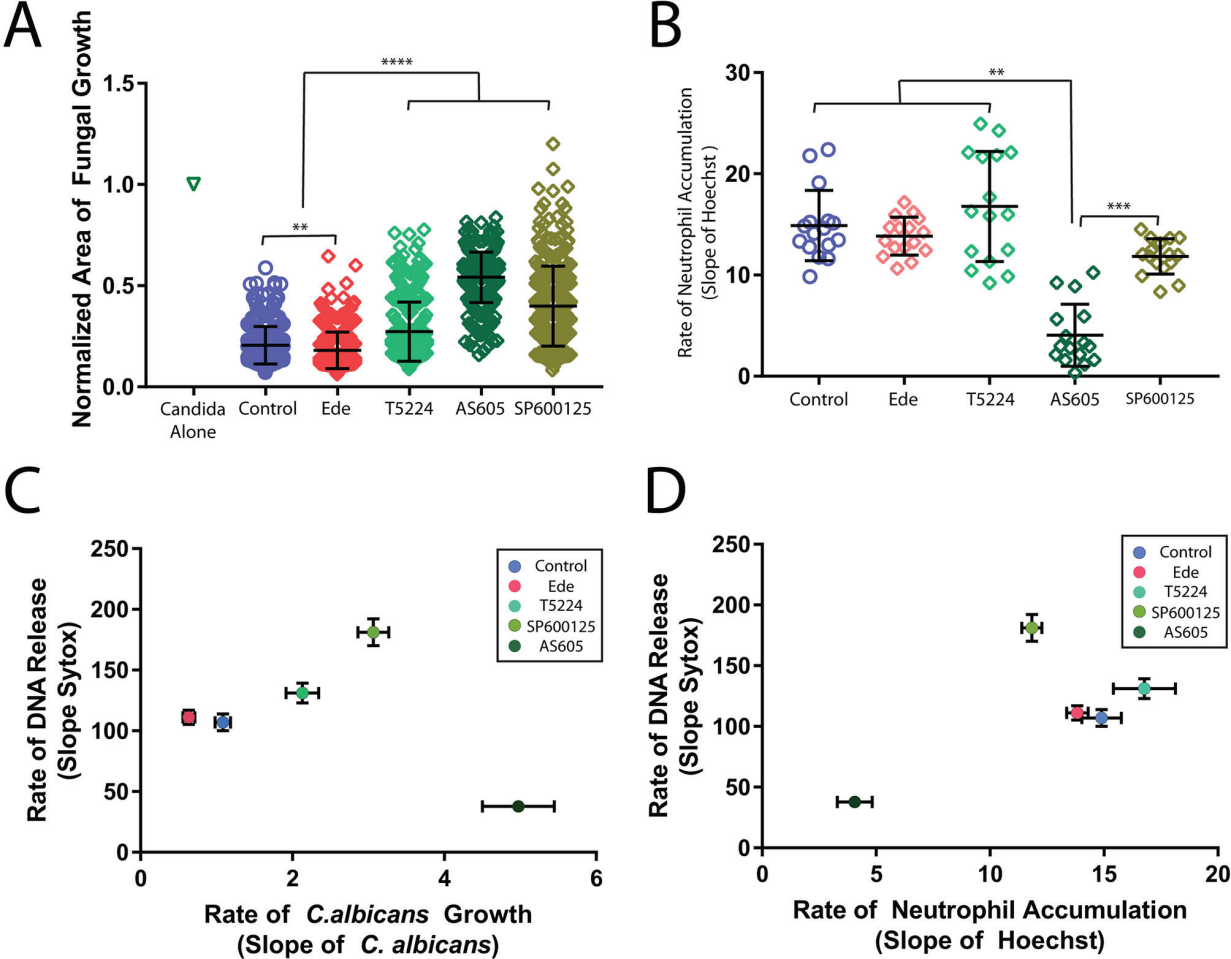
Chemical disruption of fungal restriction by neutrophil swarms. (**A**) Inhibition of PI3Ky (AS605240), Fos/AP-1 (T5224), and JNK (SP600125), but not PLC (Edelfosine), disrupt the ability of swarming to restrict *C. albicans* growth. Neutrophils were treated with the indicated inhibitor or appropriate vehicle control. The area covered by *C. albicans* after 16 h, normalized to the growth of *C. albicans* alone for the same time period, was quantified. *N* ≥ 349 swarms across four independent donors. (**B**) Neutrophils were treated with the indicated inhibitors or appropriate vehicle control and stained with Hoechst. The average fluorescent intensity was quantified at the target as an indicator of neutrophil swarm intensity. (C and D) The slope of these intensity plots was determined over the first hour. The fluorescent intensity of *C. albicans* (which expresses a far-red fluorescent protein) and of DNA release (MFI Sytox green) was quantified over the time-lapse. *N* = 16 swarms from one donor for panels C and D. Error bars represent the standard deviation for panels B and D or the standard error for panels A and C. **P ≤ 0.01, ****P ≤ 0.0001.

Building upon this finding, we probed further into the role of different signaling pathways important for neutrophil antifungal activity to see if they also played a role in swarming, targeting elements that could be acting downstream of SYK activation. We targeted phosphatidylinositol phospholipase C (PLC) with Edelfosine, PI3Kγ with AS605240, c-Fos/AP-1 with T5224, and JNK with SP600125. Edelfosine treatment slightly enhanced the overall restriction (Fig. 3A and C; Table S3). C-Fos/AP-1 inhibition by T5224 reduces the ability of neutrophils to control *C. albicans*, impairing the ability of neutrophil swarming to control fungal germination, hyphal escape, and overall fungal growth (Fig. 3A throught D; Table S3). Inhibition of JNK or PI3Kγ resulted in severe disruptions of neutrophil antifungal activity, allowing early *C. albicans* germination and hyphae escape (Fig. 3A and C; Table S3).

Interestingly, we found that PI3Kγ inhibition and JNK inhibition have distinct consequences on neutrophil swarming. While PI3Kγ inhibition reduces neutrophil recruitment to the swarm, JNK inhibition resulted in the formation of larger neutrophil swarms and release of more DNA. Surprisingly, these larger swarms did not translate to better fungal restriction, suggesting that intrinsic neutrophil antifungal mechanisms remain compromised (Fig. 3A through D; Fig. S3 and S4; Table S3). The phenotype of larger swarms and deficient fungal restriction that is seen upon JNK inhibition is similar to the phenotype we found before in chronic granulomatous disease (CGD) patients with defective ROS production (7). Therefore, we measured ROS production in the setting of the JNK inhibitor SP600125 (Fig. S4E). We found a significantly lower level of ROS compared to controls, suggesting that the changes we see in swarming dynamics during JNK inhibitor treatment are due to disruptions in ROS (Fig. S4E). All differences observed in our assays were due to changes in the neutrophils, as inhibitors did not appear to impact the growth of *C. albicans* or neutrophil viability (Fig. S5).

**Fig 4:**
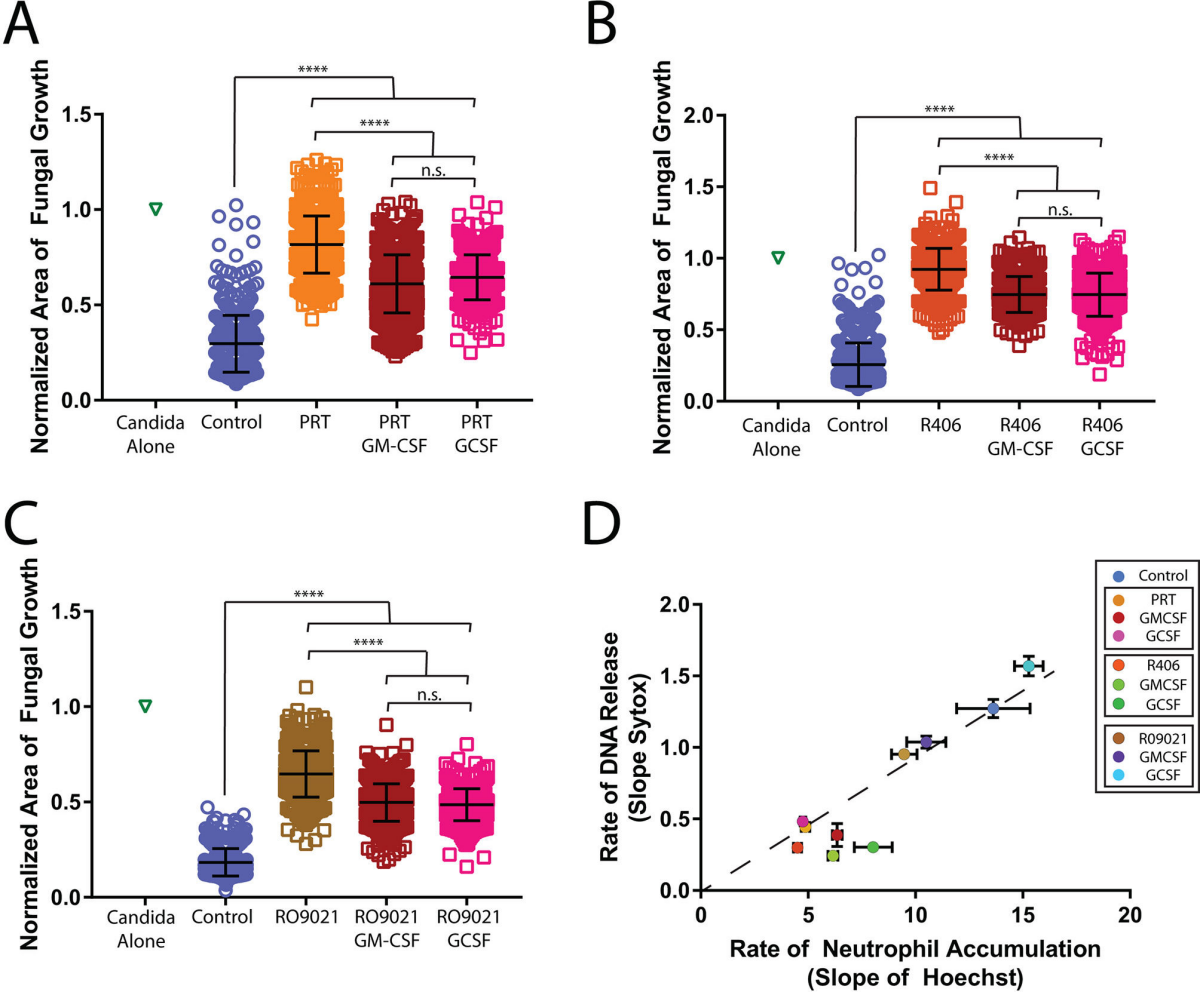
Cytokine treatment improves neutrophil function during SYK inhibition. Treatment with GM-CSF or GCSF can partially rescue fungal restriction during SYK inhibition. (**A**) Neutrophils were treated with the indicated inhibitor or inhibitor plus cytokine. The area covered by *C. albicans* after 16 h, normalized to the growth of *C. albicans* alone for the same time period, was quantified for PRT treatment. *N* ≥ 382 across at least three independent donors. (**B**) The area covered by *C. albicans* after 16 h, normalized to the growth of *C. albicans* alone for the same time period, was quantified for R406 treatment. *N* ≥ 383 across at least three independent donors. (**C**) The area covered by *C. albicans* after 16 h, normalized to the growth of *C. albicans* alone for the same time period, was quantified for RO9021 treatment. *N* ≥ 478 across at least three independent donors. (**D**) The rate of DNA release (fluorescent intensity of Sytox) and neutrophil accumulation at the swarming target (MFI Hoechst) was quantified for PRT, R406, RO9021, and cytokine priming. *N* = 16 swarms from a representative donor. Error bars represent standard deviation for panels A, C, and E or standard error for panel D. ****P ≤ 0.0001.

In our previous works, we found that the incubation of neutrophils with the cytokines GM-CSF or GCSF was able to improve swarming function in healthy neutrophils and to be able to restore some level of function in neutrophils that were inhibited in critical antifungal mechanisms or from patients with defective neutrophil function (7, 34–36). We therefore tested if GM-CSF or GCSF could overcome the defects seen during treatment with SYK inhibitors. We found that the effect of the SYK inhibitors on the ability of neutrophils to restrict fungal growth could be partially reversed by GM-CSF or GCSF (Fig. 4A through C; Fig. S6; Table S1). Interestingly, the ability to rescue neutrophil swarming function depended on the SYK inhibitor used. GM-CSF or GCSF treatment provided the greatest rescue to RO9021 and the lowest to R406-treated neutrophils. Importantly, GM-CSF or GCSF treatment did not restore control of fungal restriction to the level of control neutrophils, demonstrating the critical impairment of neutrophil antifungal interactions by the three SYK inhibitors tested. Cytokine treatment also did not significantly boost swarming in any treatment except one case (Fig. 4D; Fig. S7; Table S2). Similarly, NET responses did not appear to be as significantly changed by GM-CSF or GCSF treatment (Fig. 4D and 7; Table S2). Combination treatment of GM-CSF and GCSF provided a slight enhancement to healthy neutrophils but did not provide any significant additional enhancement to SYK-inhibited neutrophils when compared to treatment of GM-CSF or GCSF individually (Fig. S8a through c). Surprisingly, combination therapy actually seemed to perform worse than individual therapy at enhancing PRT or R406-inhibited neutrophils, though it still provided significant enhancement compared to inhibited neutrophils alone (Fig. S8b and c).

The restorative effect of GM-CSF and GCSF during treatment with SYK inhibitors occurred without boosting swarming or DNA release. This observation suggests that these cytokines act by directly augmenting neutrophil-killing mechanisms. To test this hypothesis, we conducted direct killing assays with neutrophils treated with SYK inhibitors and cytokines (Fig. S9). In line with our swarming results, we found that the killing of *C. albicans* is significantly decreased by treatment with R406 (3 ± 2% vs. 59 ± 15%, *P* < 0.0001) and RO9021 (13 ± 10% vs. 59 ± 15%, *P* < 0.0001) as compared with the vehicle control. Inhibition with R406 decreased killing significantly more than inhibition with RO9021 (3 ± 2% vs. 13% ± 10%, *P* < 0.001), which supports the differences we see with our swarming results. While priming with cytokines did not rescue the effect of the R406, priming with GM-CSF significantly improved killing in neutrophils inhibited with the RO9021 (13 ± 10% vs. 25 ± 12%, *P* < 0.05), demonstrating that cytokine treatment restores some direct neutrophil killing function (Fig. S9).

## DISCUSSION

**Fig 5:**
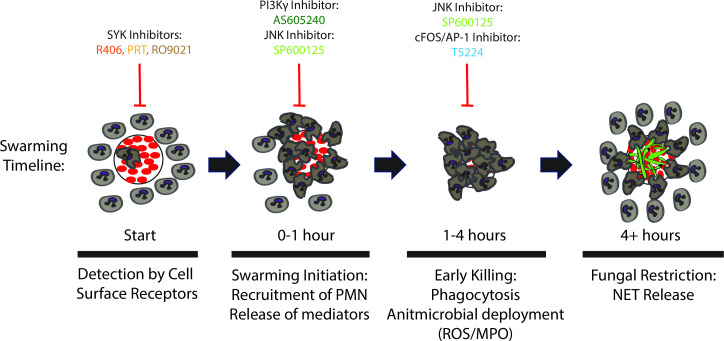
Overview of swarming of swarming steps and their inhibition. An overall timeline of swarming events impacted by each inhibitor is outlined. Based on known SYK functions and the results from this work, SYK inhibition completely disrupts swarming initiation and prevents subsequent steps from occurring. PI3Ky inhibition disrupts neutrophil locomotion and prevents swarming from occurring. JNK inhibition impacts swarming initiation, changes swarming dynamics, and results in larger swarms; however, the larger swarms after JNK inhibition are ineffective at fungal killing. AP-1 inhibition does not visibly disrupt swarming dynamics but results in a minor deficit in fungal killing. Taken together, these results provide initial insights into the molecular pathways involved in the swarming response to fungal clusters, expanding our understanding of swarming biology.

We measured the effect of various SYK inhibitors on human neutrophil swarming by employing an *in-vitro* assay that quantifies neutrophil swarming and antifungal mechanisms that help contain the growth of *C. albicans*. We show, in human neutrophils, that exposure to three SYK inhibitors completely abrogates neutrophil swarming against *C. albicans*. The effect mirrors the phenotype reported earlier in SYK knockout mouse neutrophils, suggesting that SYK signaling is critical for swarming and restricting the growth of fungi. We also show that JNK and PI3Ky signaling play vital and distinctive roles during human neutrophil swarming against *C. albicans* clusters (Fig. 5). Moreover, we show that GCSF and GM-CSF-cytokine priming partially reverses the functional deficit of human neutrophils exposed to SYK inhibitors and restores their antifungal restriction abilities.

In recent years, several molecular mechanisms involved in the function and regulation of neutrophil swarming have been revealed with the help of novel *in vitro* assays and *in vivo* animal models (3–7). The coordinated activities of neutrophils during swarming are particularly relevant to the defense against fungi, where hyphae and other enlarged morphologies prevent individual neutrophils from phagocytosing and clearing the microbes (7, 9). Our observations of the effect of three SYK inhibitors on the ability of neutrophils to migrate toward swarms and the ability to release DNA at the target, are consistent with a global importance of SYK signaling in antimicrobial responses. Our observations are also consistent with the results in mouse neutrophils, showing that interfering with SYK, through chemical inhibitors or molecular manipulation abrogates swarming (18, 39).

The defects induced by SYK inhibitors are likely multi-layered. First, there is a clear failure to recruit neutrophils to initiate and perpetuate a swarm. This early defect could be due to a failure of pioneer neutrophils to appropriately recognize the fungi and trigger the release of recruitment mediators, such as LTB_4_. The effect is consistent with reports showing that SYK is critical for the signaling downstream of many C-type lectin pattern recognition receptors known to recognize fungal cell wall components (12, 13). SYK-deficient neutrophils have been found to not have chemotaxis defects in response to existing LTB_4_ (and other chemokines) gradients, which supports that the defects shown here are largely due to a failure to recognize fungi and initiate antifungal responses, including swarming (40). Second, in keeping with this upstream role in swarm initiation, SYK inhibition has been shown to reduce the release of recruitment mediators like LTB_4_ and IL-8, which are critical for the exponential phase of swarming (14, 41). Finally, those few neutrophils that arrive at the fungal targets are less effective at pathogen containment and elimination due to the role of SYK signaling in activating essential microbial-killing responses (12–15, 18).

Our results show similarities in the alteration of neutrophil swarming phenotype in the presence of all three SYK inhibitors. The human neutrophil swarming phenotype alterations also align with those found in mouse neutrophils genetically deficient in SYK. Taken together, these findings suggest that SYK does play a critical role in human neutrophil swarming and antifungal function (18). Interestingly, the three SYK inhibitors reduce human neutrophil swarming function and antifungal control by different degrees. R406 had the biggest impact, consistent with known off-target effects, which may simultaneously disrupt several neutrophil functions (42). This highlights our assay’s sensitivity and dynamic range, as we can detect differences between multiple drugs that all have the same primary target. As with all chemical inhibitors, it is also possible that off-target effects may contribute to these differences. The changes we see may be due to one of these off-target impacts and not the primary target. Our results here must be interpreted with this caveat in mind.

Building on SYK’s apparent critical role in regulating swarming in human neutrophils, we leveraged our assay to probe further the molecular mechanisms of swarming. We tested the role of possible downstream mediators like JNK, PI3Ky, c-Fos/AP-1, and PLC. We found that JNK and PI3Ky signaling played significant roles in effective antifungal swarming responses. PI3Ky inhibition resulted in deficient swarming/neutrophil accumulation and a significant deficit in the restriction of fungal growth. These results are consistent with the published role of PI3Ky contribution to the directed migration of neutrophils to inflammatory stimuli (43–45). While the defect in migration of neutrophils to the target is likely the primary reason for the swarming defects seen with this inhibitor, PI3Ky has also been implicated in killing unopsonized *C. albicans* and in NETosis, which could further contribute to an inability to restrict fungi (17, 46).

JNK signaling has been found to regulate chemotaxis, ROS generation, and phagocytosis against microbes and has also been shown to be involved in helping epithelial cells sense *C. albicans* (47, 48). JNK inhibition also compromised the ability of neutrophils to restrict fungal growth in our swarming assay. However, the mechanism underlying these defects differs from that of PI3K, as JNK-inhibitor-treated neutrophils consistently formed larger swarms. The phenotype of large but ineffective swarms matches that observed in samples from CGD patients and in response to NADPH oxidase disruption (7). Interestingly, treatment with JNK inhibitors has been shown to reduce ROS production in LPS or *S. aureus* stimulated neutrophils (29, 48), a finding we confirmed here. This suggests that the phenotype we see could be due to ROS inhibition and adds further evidence that ROS likely plays a role in regulating swarm dynamics. JNK signaling has also been linked to the induction of iNOS expression, so disruption of this could also impact the ability of neutrophils to restrict fungi (49).

Inhibition of c-Fos/AP-1 also resulted in defects to the restriction of fungal growth, though neutrophils displayed normal recruitment profiles during swarming, suggesting their signaling importance is in relation to direct antimicrobial functions. This defect was smaller than that seen for JNK or PI3ky inhibition, suggesting that c-Fos/AP-1 signaling plays a less important role. c-Fos/AP-1 inhibition has been shown to decrease immune responses, including inflammatory cytokine production, and treatment with T5224 reduces inflammatory damage in models of arthritis and other models like acute kidney injury (50–52). AP-1 activation has also been seen in epithelial and endothelial cells in response to *C. albicans*, resulting in cytokine release and neutrophil recruitment (47, 53). Further studies will determine the specific molecular mechanisms by which c-Fos/AP-1 inhibition influences antifungal function directly in neutrophils.

Edelfosine-mediated inhibition of PLC resulted in a slight enhancement of *C. albicans* clearance, with no other obvious impact on swarming dynamics. This suggests PLC does not play a critical role in the signaling that mediates swarming behavior. Edelfosine is also known to act as an agonist for platelet-activating receptors, which could prime neutrophils and explain the minor improvements to *C. albicans* clearance that we observe (54–56). Taken together, these results highlight greater molecular detail into the pathways that are important for the swarming response and neutrophil antimicrobial function against fungal clusters. Additionally, our results demonstrate that our assay can identify and differentiate between pathways that impact chemotaxis (like PI3Kγ), antifungal function (c-Fos/AP-1), or those that appear to be involved in both (JNK, SYK). Our assay is well-positioned to continue elucidating the molecular details critical for effective swarming against fungal pathogens.

Of potential importance for patients experiencing side effects from SYK inhibitors, we show that treatment with the cytokines GCSF or GM-CSF allows us to rescue swarming and antifungal function during SYK inhibition. This rescue is partial and does not seem to involve significantly restoring neutrophil mobility to swarm toward the target. This result is different from previous data showing that GM-CSF or GCSF treatment could partially restore or boost swarming motility during MPO or ROS inhibition (7). These observations suggest that the GCSF or GM-CSF cytokines are most likely enhancing the fungal killing mechanisms during swarming. In support of this, direct neutrophil killing assays revealed the GCSF treatment enhances *C. albicans* killing during SYK inhibition, though only during treatment with R09021. GCSF treatment could not significantly enhance killing during inhibition by R406 in this assay, which showed a more severe defect than R09021, in agreement with our swarming results. Interestingly, the benefits of GCSF priming seem to be more evident in swarming than in the planktonic conditions of the killing assay, as GCSF and GM-CSF showed significant enhancement to the fungal restriction during swarming that was not seen in the killing assay (i.e., for R406). This could be because swarming involves communications between neutrophils that amplify their response, and even minor enhancement could be amplified to significant differences during swarming. While GCSF is typically thought of in its role in granulopoiesis, it has been shown to enhance ROS production, phagocytosis, and microbial killing in other contexts, which could explain how it enhances antimicrobial function during swarming even during SYK inhibition (57–63). Interestingly, combining GM-CSF and GCSF did not offer any additional benefit compared to treatment with just GM-CSF or GCSF, suggesting that they may use overlapping pathways when rescuing SYK inhibition. Further work will elucidate the molecular mechanisms CSFs can directly rescue neutrophil function.

Due to its major role in B lymphocyte signaling, SYK signaling has been highlighted as a therapeutic target and has shown beneficial results in models of autoimmune diseases like rheumatoid arthritis, systemic lupus erythematosus, and thrombocytopenic purpura as well as in the treatment of B-cell malignancies (14, 24–26, 64). However, these exciting benefits are dampened in clinical trials by the increased risk for infections in patients treated with SYK inhibitors (2, 27). Even when viewed through the complicating lens of possible off-target effects, it is clear that treatment with these SYK inhibitors causes severe disruptions to neutrophil swarming and antifungal function by some mechanism. Therefore, a more complete understanding of neutrophil antifungal functions could present a path to design therapies that boost neutrophil antimicrobial function without counteracting the therapeutic benefits of SYK inhibition. Our study suggests that the neutrophil disruptions triggered by SYK inhibitors could be partially restored by treatment with GM-CSF or GCSF, though the exact mechanisms by which this occurs remain uncharacterized. The feasibility of boosting neutrophil antimicrobial function is further supported by a recent case study when GCSF administrations significantly reduced the number of infections they experienced (34). GCSF and GM-CSF also helped restore swarming function in cirrhotic liver, solid organ, and stem cell transplant patient neutrophils *in vitro* (35, 36). Thus, elucidating the role of SYK and other signal transduction elements during neutrophil swarming could have clinical benefits (1, 11, 16). Toward this goal, leveraging new assays like the swarming arrays will be critical for characterizing these pathways to design new therapeutic strategies to address defective neutrophil functions and develop therapies to combat autoimmune disease with fewer side effects.

## SUPPLEMENTAL MATERIAL

Supplemental material spectrum.02549-21-s0001.docx Tables S1 to S3; Fig. S1 to S9.

ASM does not own the copyrights to Supplemental Material that may be linked to, or accessed through, an article. The authors have granted ASM a non-exclusive, world-wide license to publish the Supplemental Material files. Please contact the corresponding author directly for reuse.
