# Supplementary material for: Spleen tyrosine kinase inhibitors disrupt human neutrophil swarming and antifungal functions
Source: Microbiol Spectr. 2024 Nov 27;13(1):e02549-21. doi: 10.1128/spectrum.02549-21 (PMC11705959; doi:10.1128/spectrum.02549-21)
Supplement: Supplemental material — Tables S1 to S3; Fig. S1 to S9. [file spectrum.02549-21-s0001.docx]

**Supplementary Data**

**Spleen Tyrosine Kinase Inhibitors Disrupt Neutrophil Swarming and Antifungal Functions**

**Authors:** Alex Hopke,^1,2,3^ Adam L. Viens^4^, Natalie J. Alexander^4^ , Seok Joon Mun^1^, Michael K. Mansour,^4^ Daniel Irimia^1,2,3*^

**Affiliations:**

^1^BioMEMS Resource Center, Massachusetts General Hospital, Boston, MA, 02129, USA

^2^Harvard Medical School, Boston, MA, 02115, USA

^3^Shriners Hospital for Children, Boston, MA, 02114, USA

^4^Division of Infectious Diseases, Massachusetts General Hospital, Boston, MA, 02114, USA

*Correspondence to:

Daniel Irimia: ([dirimia@mgh.harvard.edu](mailto:dirimia@mgh.harvard.edu))

Tables:

**Table 1: SYK Inhibition Results in Deficient Fungal Restriction**. The average area of fungal growth, time to germination (minutes), and time to hyphal escape (minutes) during SYK inhibition or during SYK inhibition with GM-CSF or GCSF treatment is shown. Data shown is summarized from Figure 1. Figure 4 and Supplemental Figure 5.


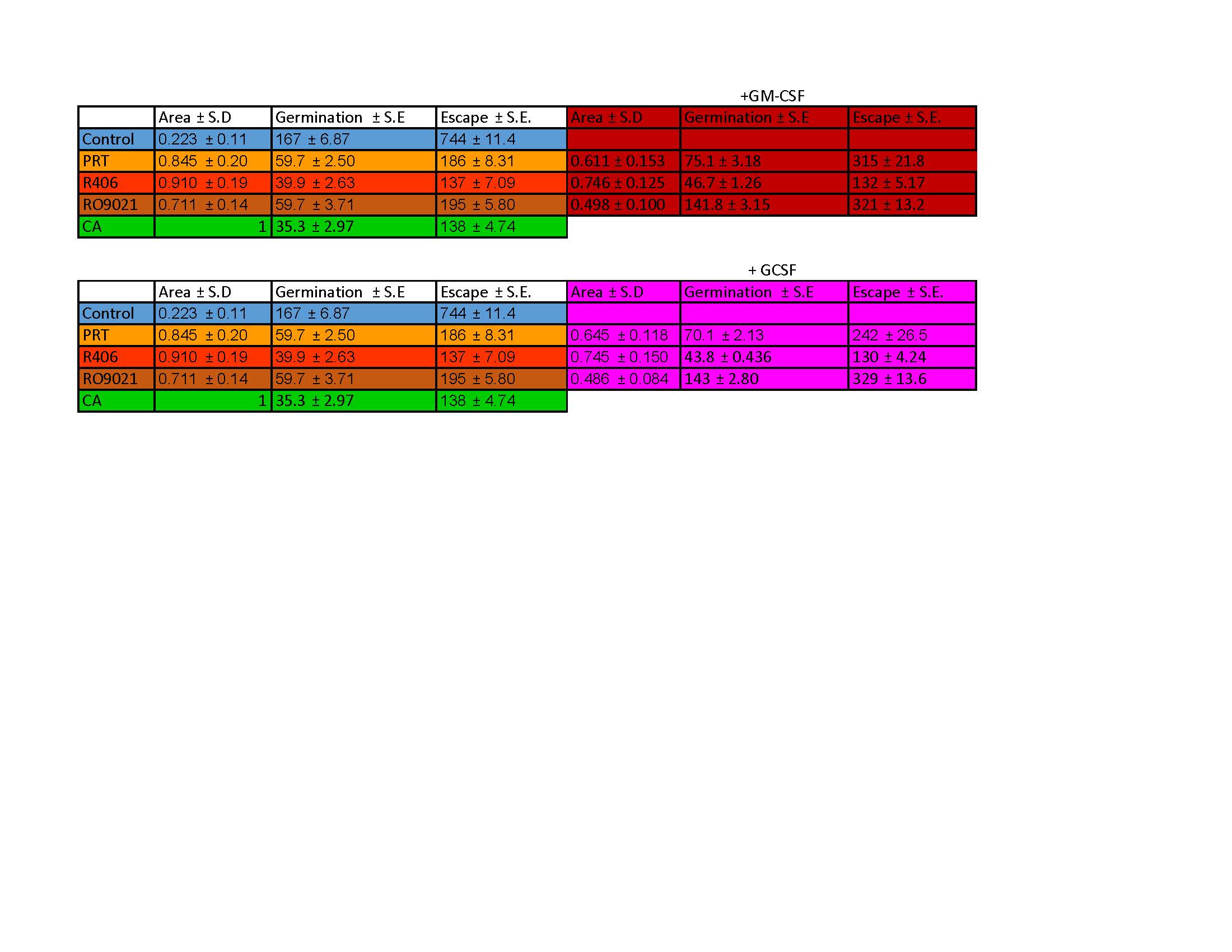


**Table 2: SYK Inhibition Results in Defective Swarming and NET Release.** The average rate of neutrophil accumulation during the first hour of the assay or the average rate of NET release from 6-12 hours in the assay during SYK inhibition and also for SYK inhibition with treatment by GM-CSF or GCSF. Data shown is summarized from Figure 2, Figure 4, Supplemental Figure 6).


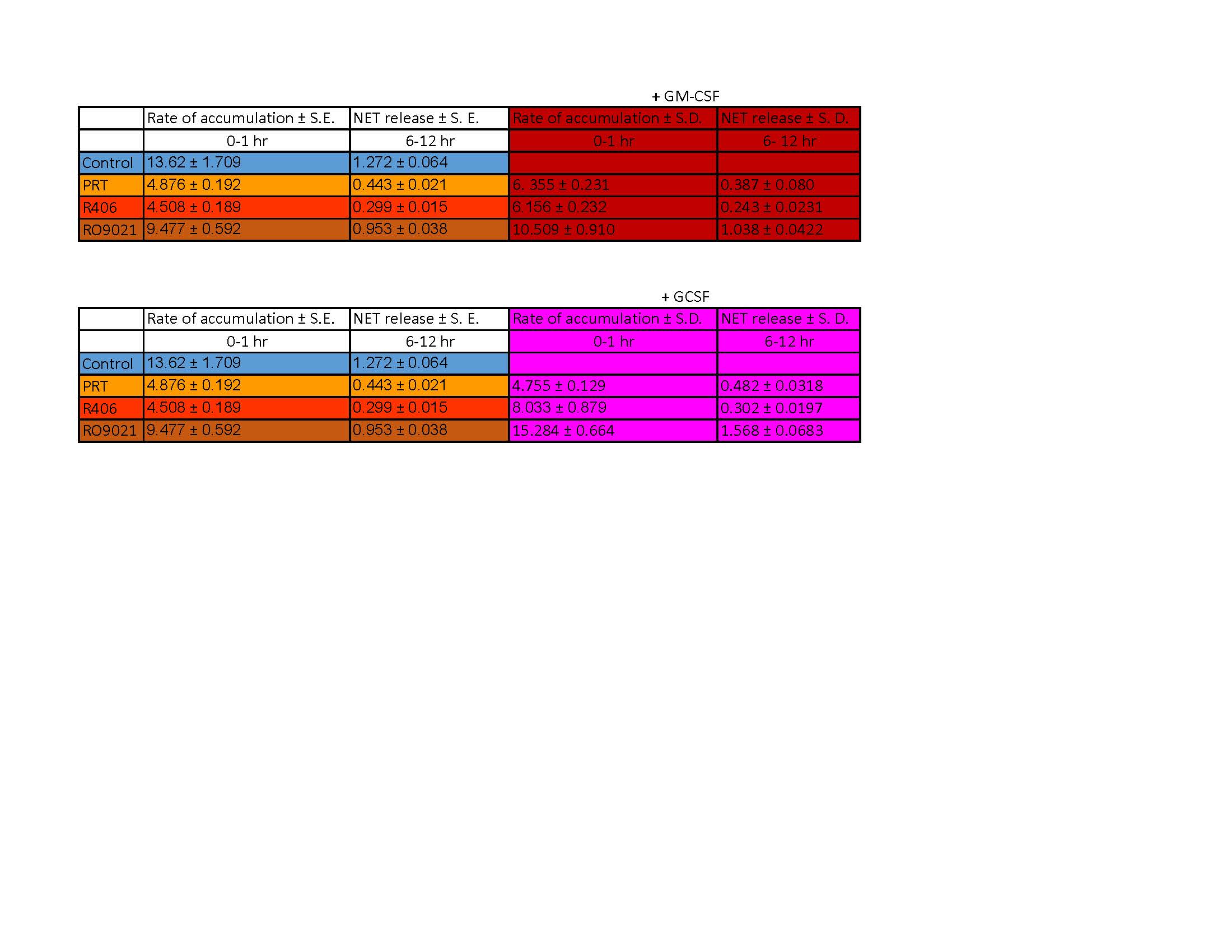


**Table 3: Impact of Inhibitors on Fungal Restriction**

The average area of fungal growth, time to germination (minutes), time to hyphal escape (minutes), as well as the average rate of neutrophil accumulation during the first hour of the assay or the average rate of NET release from 6-12 hours in the assay for the data shown in Figure 3 and Supplemental figure 2 is summarized.


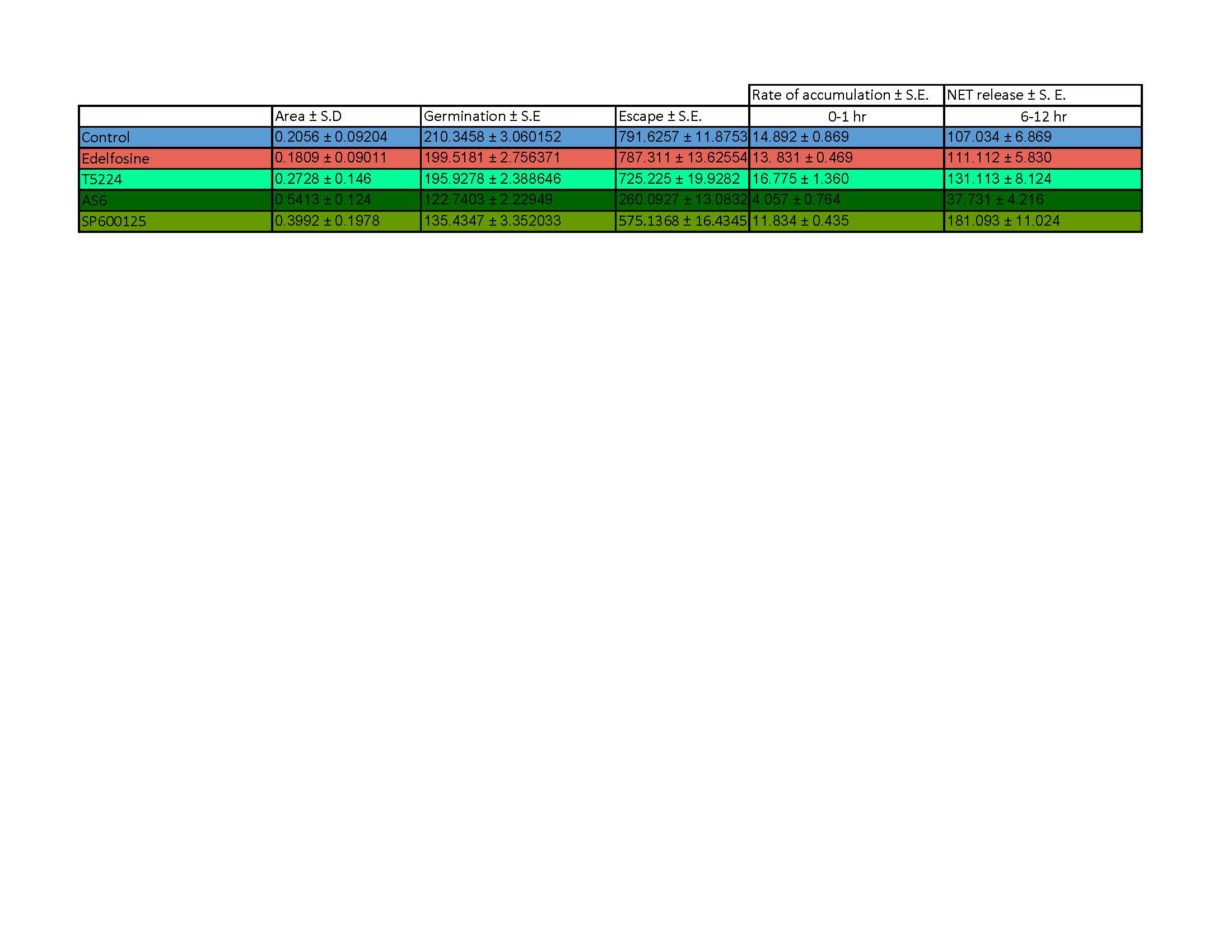


**Supplemental Figure 1: SYK Inhibitors Inhibit Neutrophil Swarming**


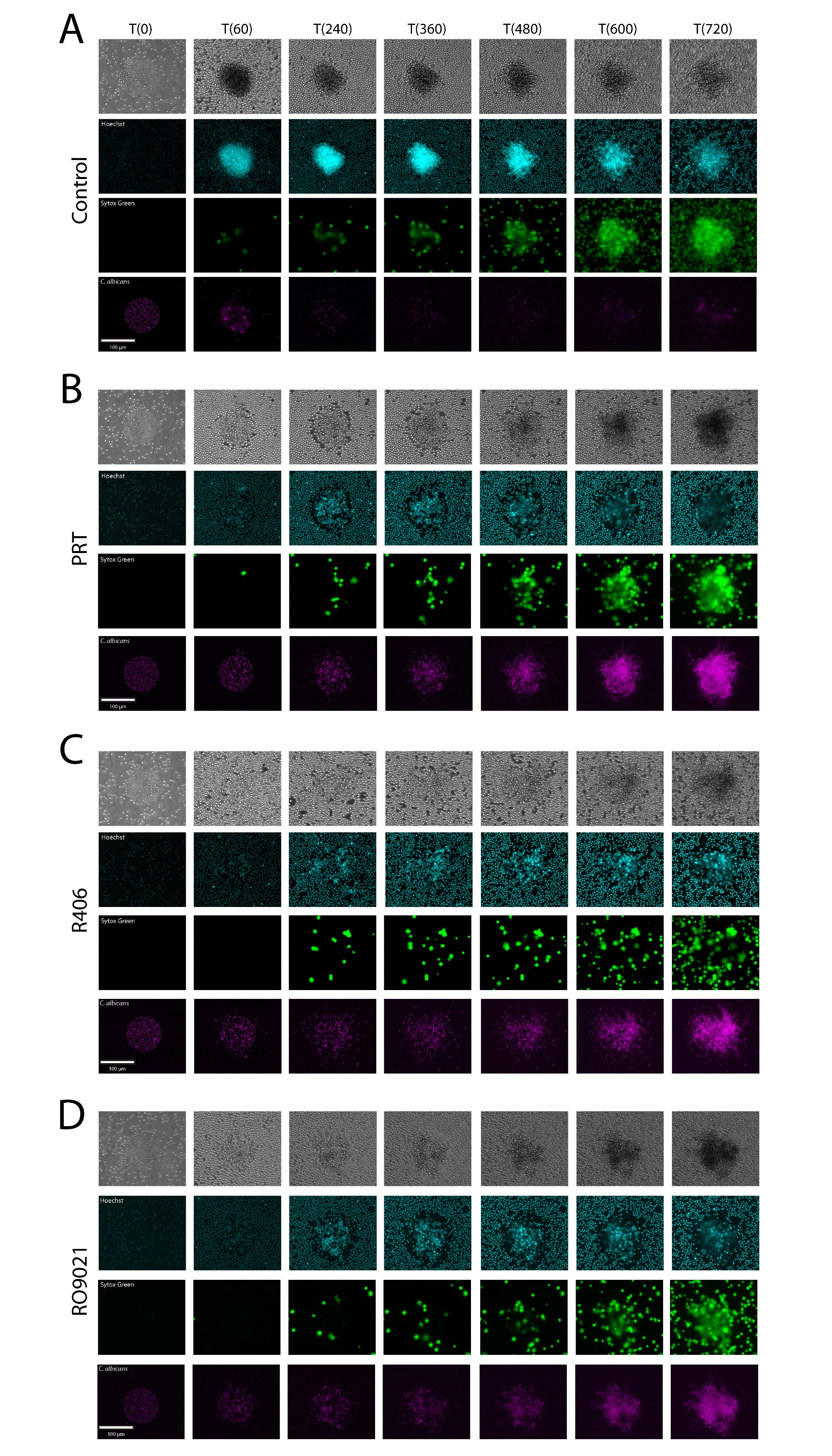


Human neutrophils were treated with the indicated SYK inhibitors or an appropriate vehicle control. Representative images from timelapse experiments show neutrophils (Hoechst), NET release (Sytox Green) and *C. albicans* growth (Far Red), along with the brightfield during each treatment. Scale bar represents 100 µm.

**Supplemental Figure 2: Quantitation of NET Release by Nuclei Decondensation**


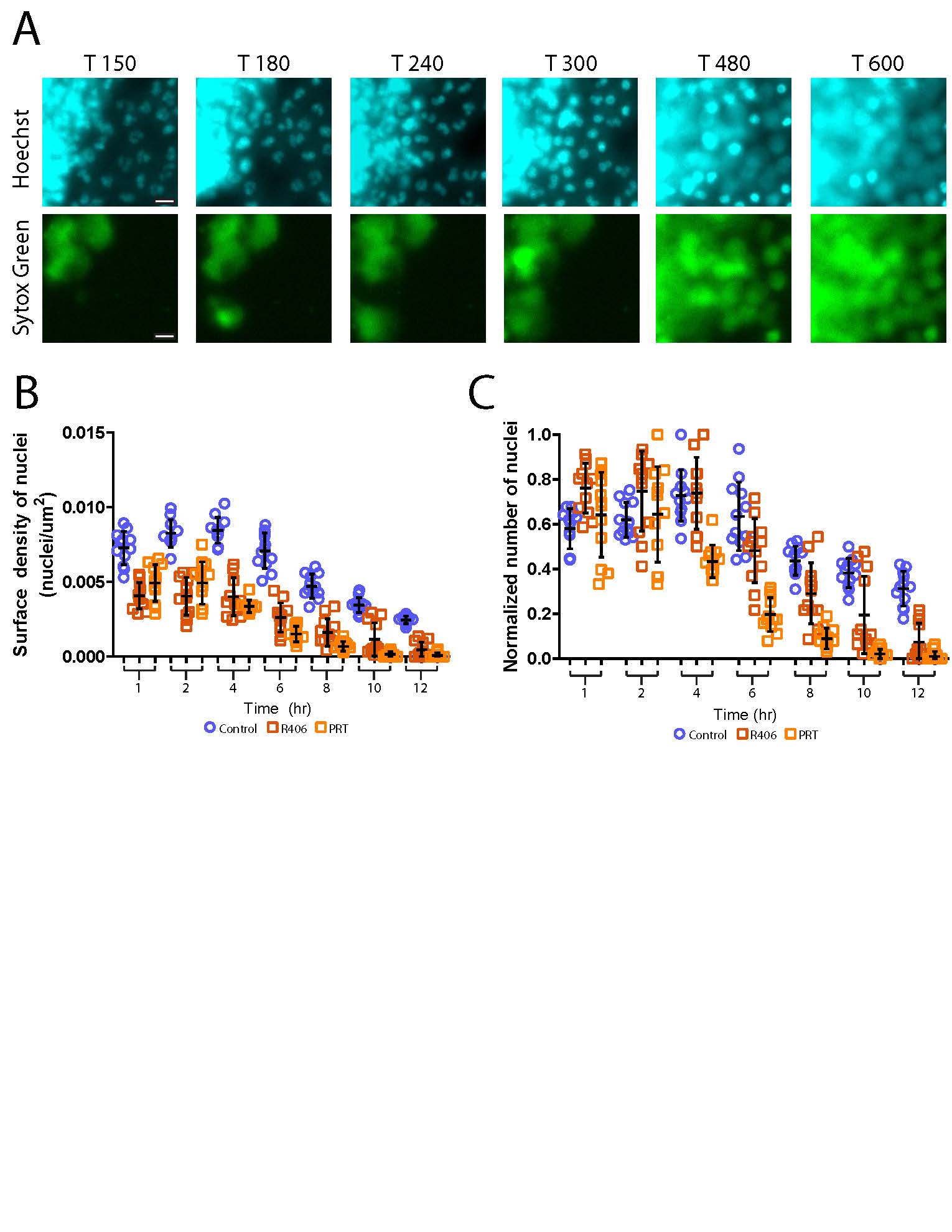


Human neutrophils were incubated with the indicated inhibitor or vehicle, stained with Hoechst, and then added to the swarming assay and visualized by timelapse microscopy. (A) Representative images, showing the progression of nuclei from condensed to diffuse over time (minutes) at the edge of the swarm, are shown with Hoechst (blue) and Sytox Green (green) channels. Scale bar is 10 µm. (B) Individual condensed nuclei were quantified at each *C. albicans* cluster over time. Results were normalized against the area of the swarm (for control) or against the area of the original target cluster (for R406 and PRT). (C) Alternatively, to normalize against the different number of neutrophils involved, the number of condensed nuclei was normalized against the peak number of nuclei for that condition. N=12 swarms for each condition across three donors.

**Supplemental Figure 3: Chemical Disruption of Neutrophil Swarming.** Inhibition of PI3-Kγ disrupts the ability neutrophils to swarm while disruption of JNK results in increased NET release. (A). The fluorescent intensity of *C. albicans* (which expresses a far red fluorescent protein) was quantified over the timelapse. (B). The rate of change of *C.albicans* fluorescent intensity from 7-13 hours was greatest for AS605240. N=16 swarms from 1 donor for A-B. (C). The time it took *C. albicans* yeast to germinate and for hyphae to then escape the area of the neutrophil swarm was quantified. N=48 swarms from 3 donors. (D). Neutrophils were treated with the indicated inhibitors or appropriate vehicle control and stained with Hoechst. The average fluorescent intensity was quantified at the target as an indicator of neutrophils swarm intensity. N=16 swarms from a single representative donor. (E). The average fluorescent intensity of sytox green staining at the target was quantified over the timelapse as an indicator of NET release. (F). The approximate slope of the intensity plots from hour 5-13 were determined. Error bars represent standard deviation for B,D and standard error for A, C. N=16 swarms from one donor for E-F. Error bars represent standard deviation for B, F and standard error for A, C, D, E. **p≤0.01,***p≤0.001, ****p≤0.0001.


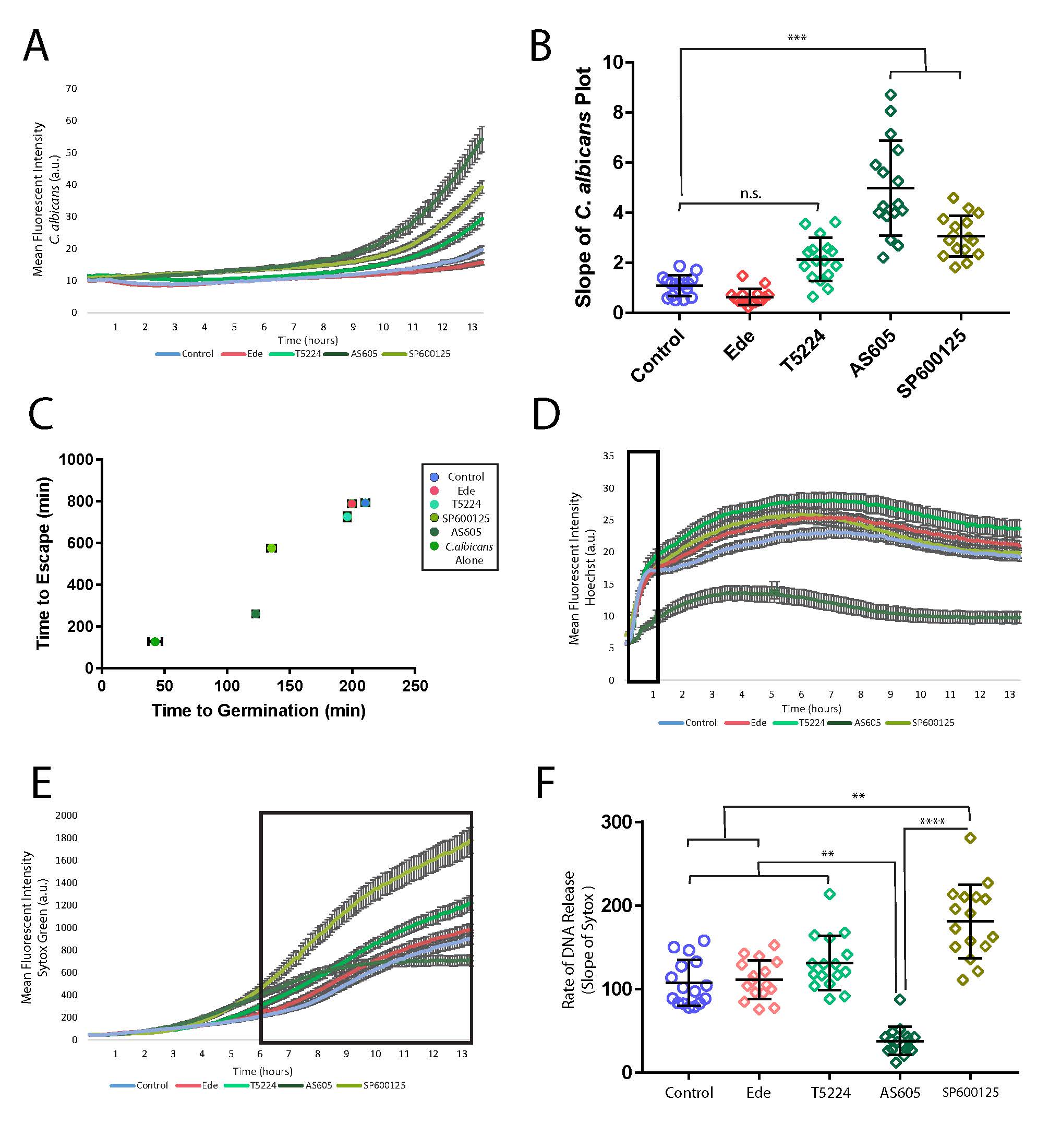


**Supplemental Figure 4: JNK Inhibition Results in Larger Swarms and Reduction of ROS**


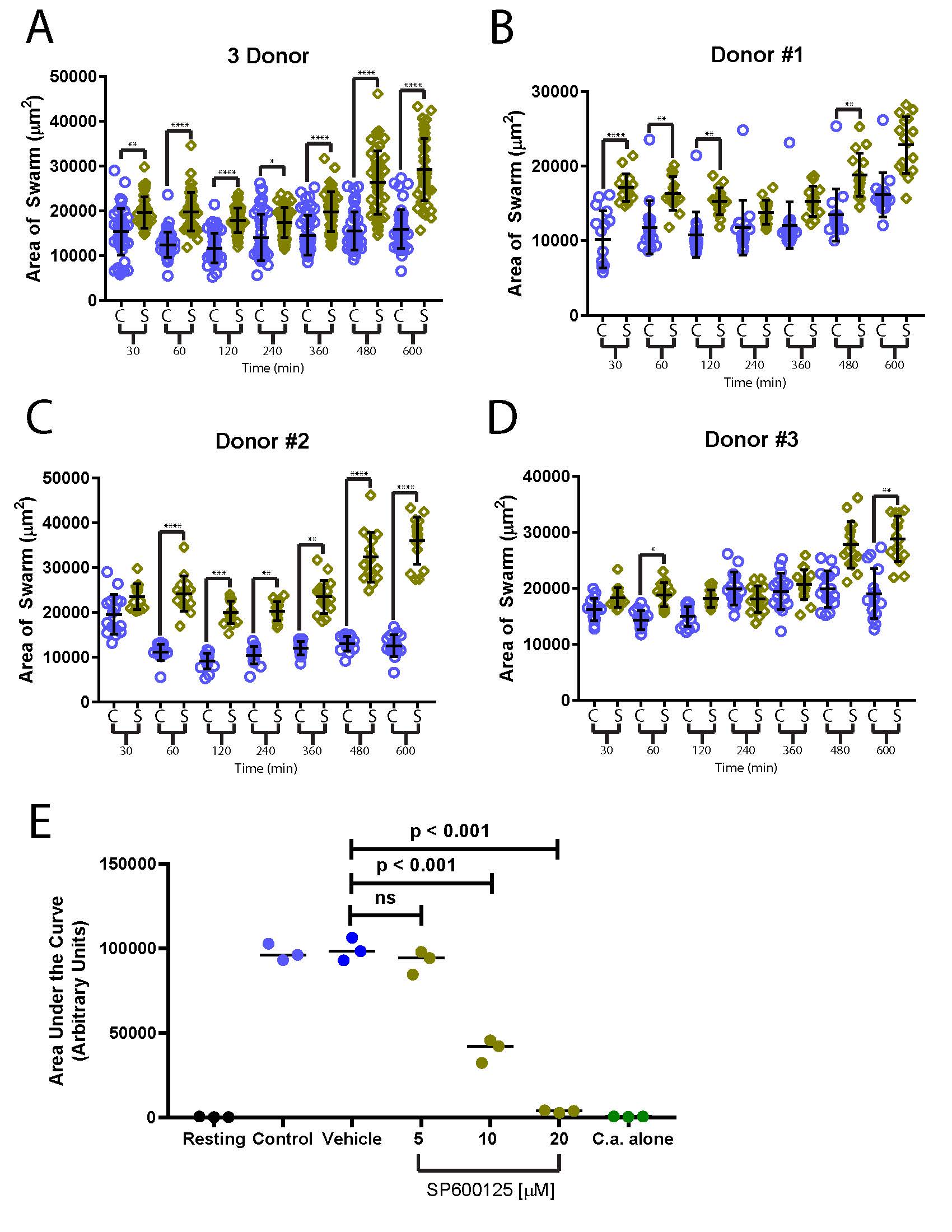


Neutrophils were treated with vehicle control (C) or JNK inhibitor SP600125 (S), run in the swarming assay against clusters of *C. albicans* and observed via fluorescent timelapse microscopy. The area of the neutrophil swarm was quantified at the indicated timepoints. The results are shown for three donors pooled (A) or each donor individually (B-D). N= 48 swarms across three independent donors for A and N=16 swarms from each individual donor for B-D. (E) Neutrophils were co-cultured with heat killed *Candida albicans* hyphae for four hours with or without a JNK inhibitor and ROS production was assessed for by luminescence in the presence of Lucigenin. The results reflect the mean ± SEM of one experiment and is a representative of 3 independent experiments. *p≤0.05, **p≤0.01, ***p≤0.001, ****p≤0.0001.

**Supplemental Figure 5: Inhibitors do not Impact Fungal Growth or Neutrophil Viability**


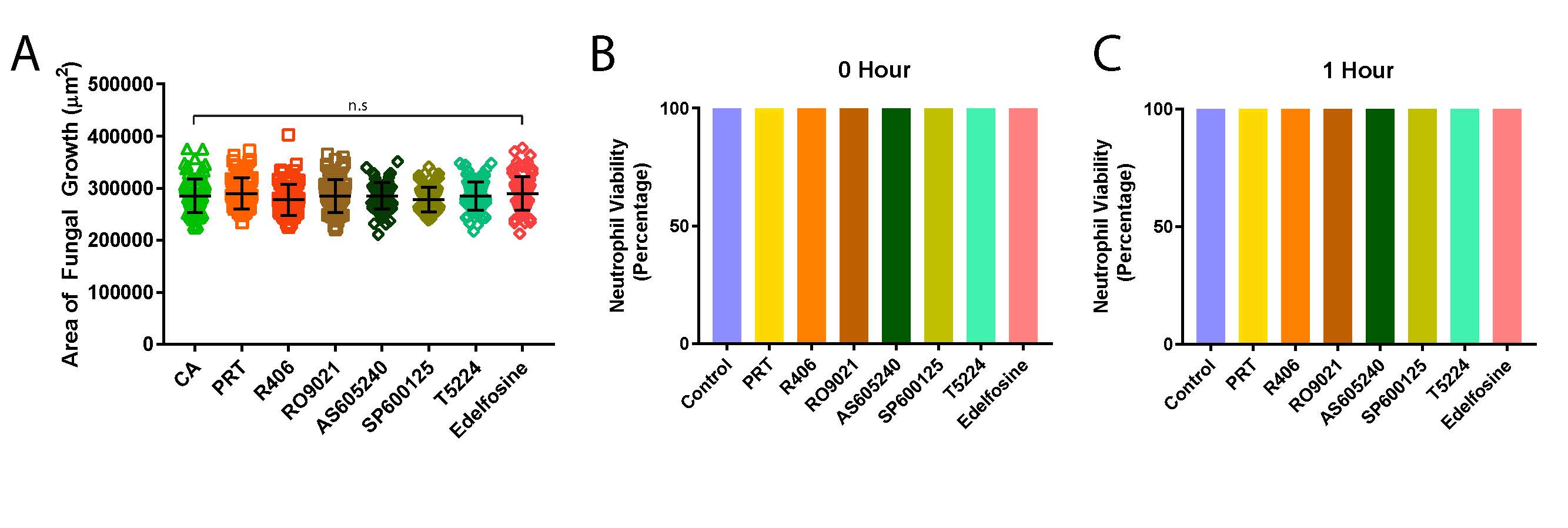


*C. albicans* clusters were grown with media only or with the indicated inhibitor included in the media. The area of fungal growth was quantified after 16 hours of growth at 37°C. N≥ 93 spots (A). Dead cells (sytox green positive) were quantified in each field of view at the very start of the assay (B) and at 1 hour (C). Viability is expressed as the percent live cells against the average number of cells in a field of view. N=8 fields of view per condition. n.s is non-significant.

**Supplemental Figure 6: Cytokine Priming can Partially Improve Fungal Restriction**


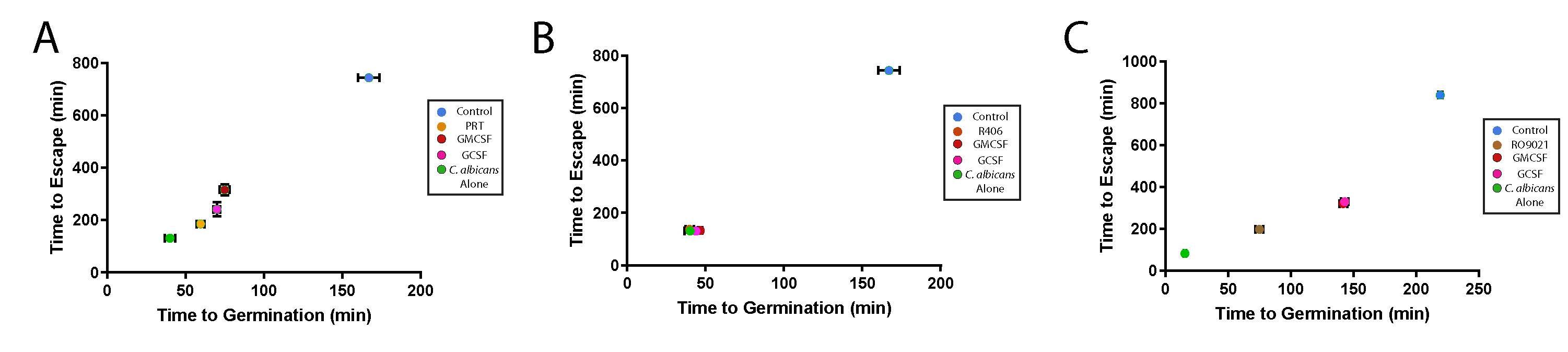


Treatment with GM-CSF or GCSF can partially rescue fungal restriction during SYK inhibition. (A). Neutrophils were treated with the indicated inhibitor or inhibitor plus cytokine. The time it took *C. albicans* yeast to germinate and for hyphae to then escape the area of the neutrophil swarm was quantified during PRT treatment. N≥ 48 swarms across at least 3 independent donors. (B). The time for *C. albicans* germination and hyphal escape during R406 treatment was quantified. N≥ 48 swarms across at least 3 independent donors. (C). The time for *C. albicans* germination and hyphal escape during RO9021 treatment was quantified. N=32 swarms across 2 independent donors.


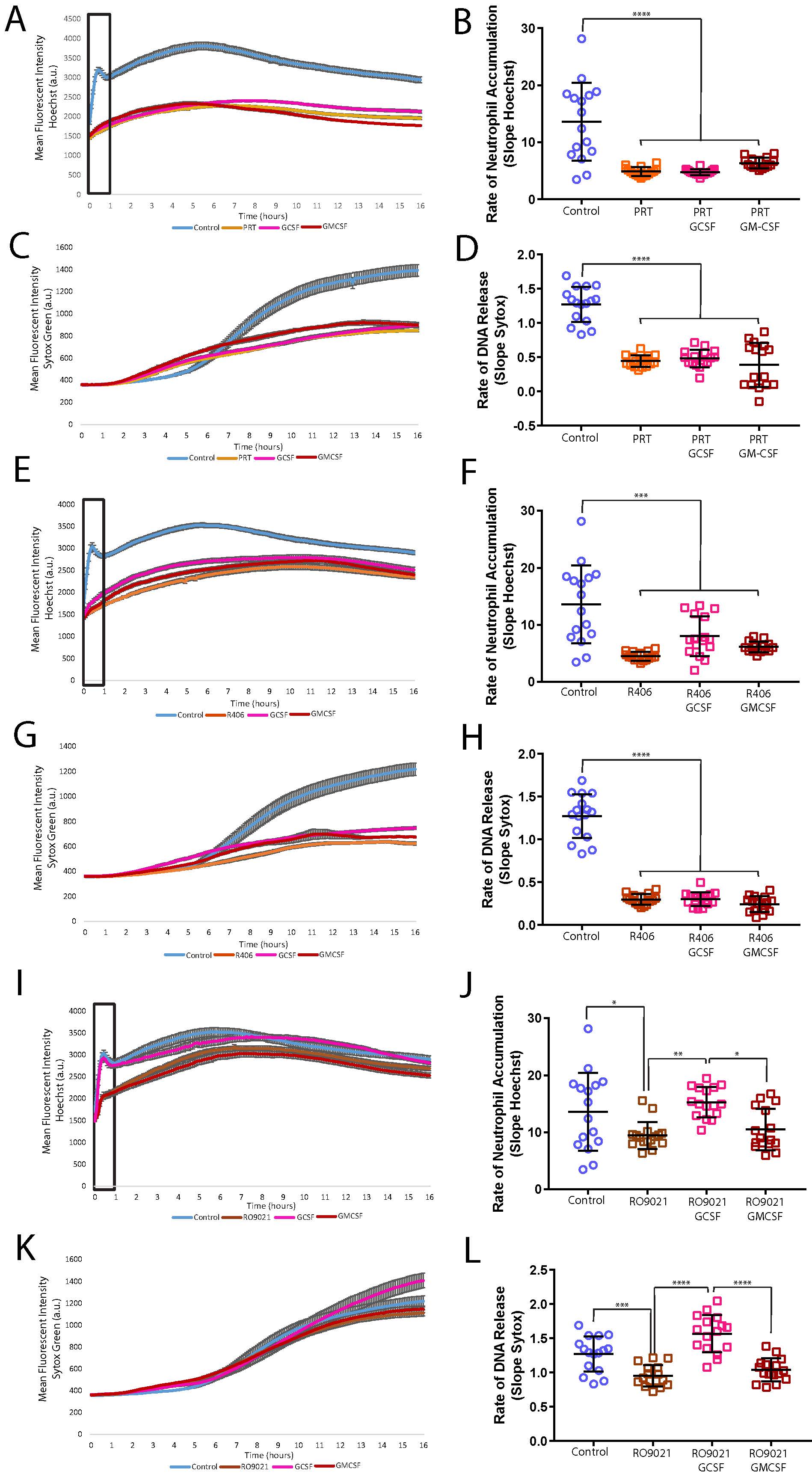


**Supplemental Figure 7: Cytokine Treatment Does Not Boosts Neutrophil Swarming.** The average fluorescent intensity was quantified at the target as an indicator of neutrophils swarm intensity for treatment with PRT+ cytokine (A), R406 + cytokine (E) or RO9021 + cytokine (I). The slope of these intensity plots was determined over the first hour (B, F, J). The average fluorescent intensity of sytox green staining at the target was quantified over the timelapse as an indicator of NET release for PRT + cytokine (C), R406 + cytokine (G) or RO9021 + cytokine (K). The approximate slope of the intensity plots from hour 5-13 were determined (D, H, L). N=16 swarms from one donor for all panels. Error bars represent standard deviation for B, D, F, H, J, L and standard error for A, C, E, G, I, K. *p≤0.05, **p≤0.01, ***p≤0.001, ****p≤0.0001.

**Supplemental Figure 8: Combination Cytokine Priming does not Improve Fungal Restriction Over Individual Cytokine Therapy**


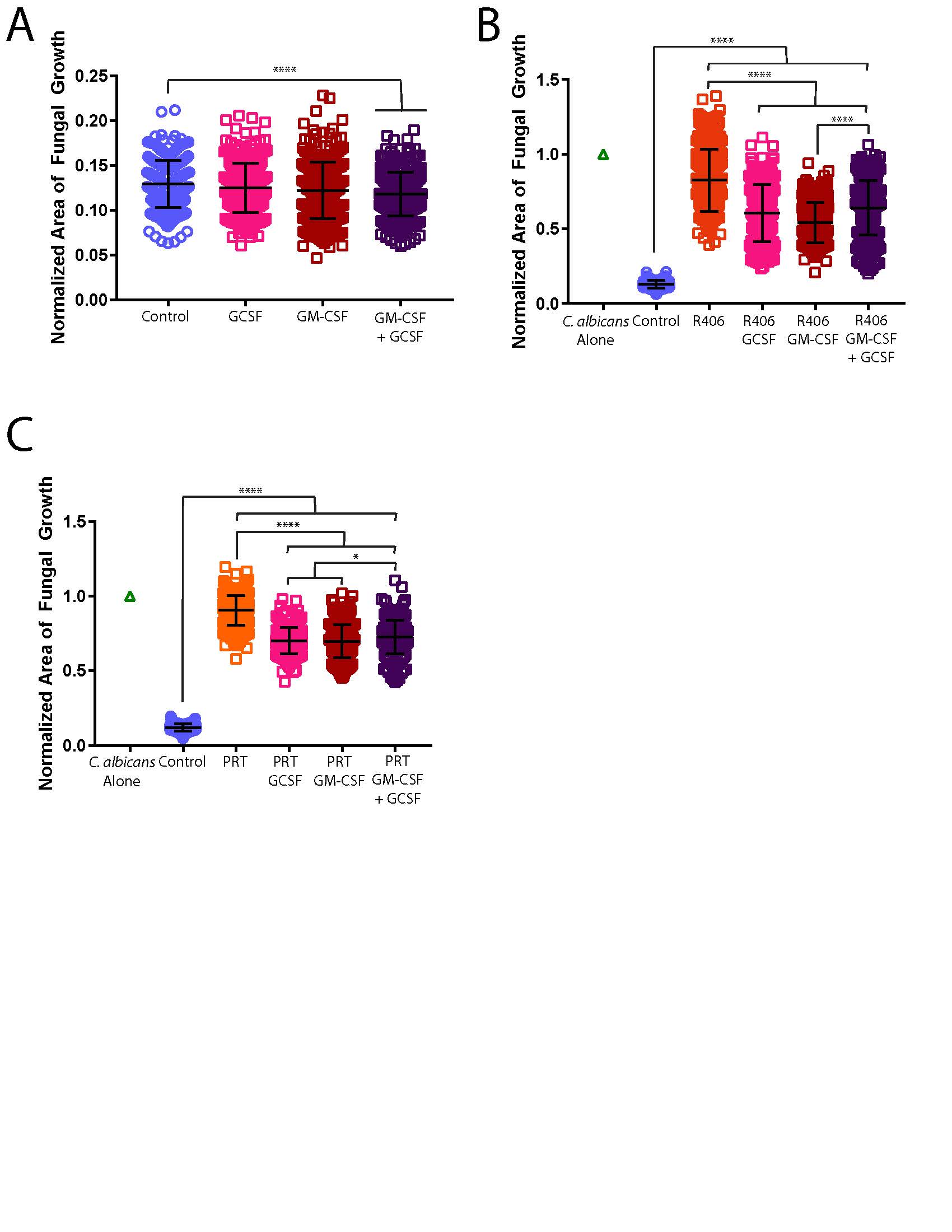


Treatment with GM-CSF or GCSF can partially rescue fungal restriction during SYK inhibition but combination GM-CSF and GCSF treatment does not provide additional enhancement. (A). Neutrophils were treated with the indicated cytokine or cytokine combination. The area covered by *C. albicans* after 16 hours, normalized to the growth of *C. albicans* alone for the same time period, was quantified. N≥288 swarms across 3 independent donors. (B). Neutrophils were treated with the indicated inhibitor (R406), cytokine or cytokine combination. The area covered by *C. albicans* after 16 hours, normalized to the growth of *C. albicans* alone for the same time period, was quantified. N≥288 swarms across 3 independent donors. (C). Neutrophils were treated with the indicated inhibitor (PRT), cytokine or cytokine combination. The area covered by *C. albicans* after 16 hours, normalized to the growth of *C. albicans* alone for the same time period, was quantified. N≥288 swarms across 3 independent donors.

**Supplemental Figure 9: Cytokine Priming Rescues Neutrophil Function in Direct Killing Assays**


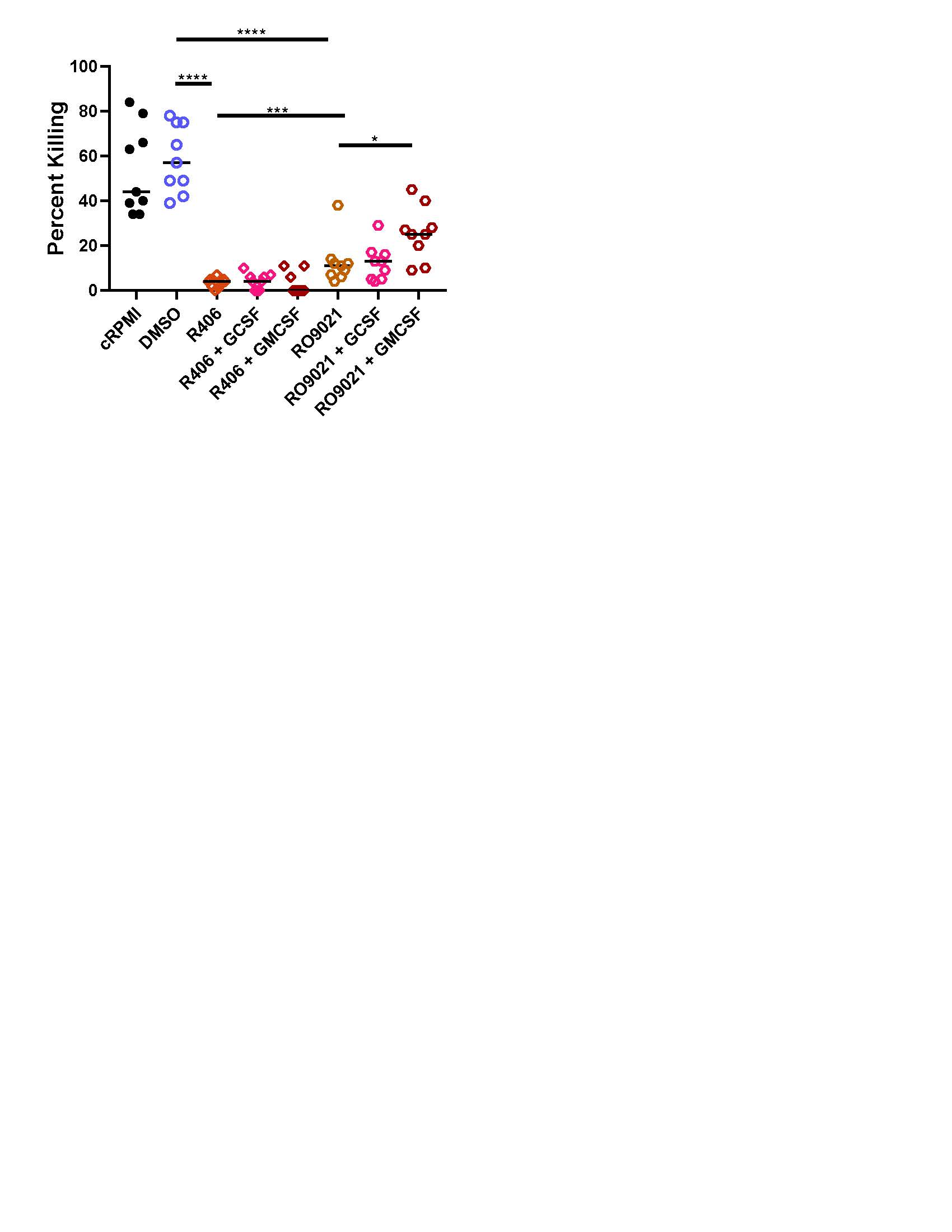


Human neutrophils were preincubated with SYK inhibitors, SYK inhibitors + cytokines, or just vehicle and then incubated with C. albicans yeast for 2 hours. Percent killing was calculated by comparing the fluorescence values from wells containing neutrophils and *Candida*, to the fluorescence of wells with *Candida* alone. Drug and cytokine treated wells were then compared to vehicle control wells (DMSO) or each other. N=9 wells pooled together across 3 independent donors. Significance was determined by a Mann-Whitney test, with significance defined as p<0.05.
